# High-Power-Density, High-Energy-Density Fluorinated Graphene for Primary Lithium Batteries

**DOI:** 10.3389/fchem.2018.00050

**Published:** 2018-03-09

**Authors:** Guiming Zhong, Huixin Chen, Xingkang Huang, Hongjun Yue, Canzhong Lu

**Affiliations:** ^1^CAS Key Laboratory of Design and Assembly of Functional Nanostructures, and Fujian Provincial Key Laboratory of Nanomaterials, Fujian Institute of Research on the Structure of Matter, Chinese Academy of Sciences, Fuzhou, China; ^2^Xiamen Institute of Rare Earth Materials, Haixi Institutes, Chinese Academy of Sciences, Xiamen, China; ^3^Department of Mechanical Engineering, University of Wisconsin-Milwaukee, Milwaukee, WI, United States

**Keywords:** fluorinated graphene, carbon fluoride, primary lithium battery, nuclear magnetic resonance, high power density

## Abstract

Li/CF_x_ is one of the highest-energy-density primary batteries; however, poor rate capability hinders its practical applications in high-power devices. Here we report a preparation of fluorinated graphene (GF_x_) with superior performance through a direct gas fluorination method. We find that the so-called “semi-ionic” C-F bond content in all C-F bonds presents a more critical impact on rate performance of the GF_x_ in comparison with sp^2^ C content in the GF_x_, morphology, structure, and specific surface area of the materials. The rate capability remains excellent before the semi-ionic C-F bond proportion in the GF_x_ decreases. Thus, by optimizing semi-ionic C-F content in our GF_x_, we obtain the optimal x of 0.8, with which the GF_0.8_ exhibits a very high energy density of 1,073 Wh kg^−1^ and an excellent power density of 21,460 W kg^−1^ at a high current density of 10 A g^−1^. More importantly, our approach opens a new avenue to obtain fluorinated carbon with high energy densities without compromising high power densities.

## Introduction

Fluorinated carbon (CF_x_) possesses a very high theoretical energy density (2,180 Wh kg^−1^ when x equals 1 for fluorinated graphite) as a cathode material for primary lithium batteries, thus has been strongly desired in many civil and military applications that require a long service-life, wide range of operating temperatures, as well as high energy densities and reliability. Fluorinated graphite has been widely investigated (Nakajima et al., 2002; Guérin et al., 2004; Zhang Q. et al., 2010). Besides graphite, other types of carbons such as carbon nanotubes, carbon nanofibers, C60, and mesoporous carbon, have also been applied for fluorination (Matsuo and Nakajima, 1996; Mickelson et al., 1998; Lam and Yazami, 2006; Yazami et al., 2007; Zhang W. et al., 2010; Fulvio et al., 2011; Guérin et al., 2012). Among these, a fluorinated mesoporous carbon (CF_0.54_) and a fluorinated coke displayed excellent performance; the fluorinated mesoporous carbon delivered a capacity of 515 mAh g^−1^ with discharge plateau of 2.75 V at a current rate of 5C (Fulvio et al., 2011), while the fluorinated coke displayed a maximum power density of about 14,400 W kg^−1^ with energy density of 500 Wh kg^−1^ (Lam and Yazami, 2006). However, the power densities of these materials are far from satisfaction because of the poor electronic conductivity of the CF_x_ materials due to the strong covalent C-F bond.

Coating of highly conductive materials, such as carbon, polypyrrole, and polyaniline on the surface of carbon fluorides is helpful to improve the rate capability (Zhang Q. et al., 2010; Groult et al., 2011; Li et al., 2016); for example, a graphite fluoride coated with polyaniline delivered an energy density of about 1,200 Wh kg^−1^ with power density higher than 10,000 W kg^−1^ at current rate of 8C (Li et al., 2016). An amazing rate performance (48,800 W kg^−1^ at 30C) was achieved by reducing the fluorine content on the surface of the carbon fluorides through hydrothermal method, which greatly improved the electronic conductivity (Dai et al., 2014). However, the hydrothermal reaction would be restricted for practical application because it could introduce a large amount of hydroxyl group, jeopardizing the calendar life of the Li/CF_x_ battery.

Fluorinated graphene, as a two-dimensional (2D) material, can shorten the diffusion path of lithium ions, which is helpful for rapid transfer of lithium ions (Zhang S. S. et al., 2009; Feng et al., 2016), thus opening an alternative avenue to chase excellent rate capability. Until now the best fluorinated graphene reported in the literature only can work at 5C, gaining a capacity of 356 mAh g^−1^ (GF_0.47_) (Damien et al., 2013; Meduri et al., 2013; Zhao et al., 2014; Feng et al., 2016). Therefore, uncovering the reasons hindering fluorinated graphene from achieving excellent rate capability is highly desired.

In this study, a fluorinated multilayered graphene (GF_x_) was prepared by a direct gas fluorination of RGO instead of graphene oxides (Damien et al., 2013; Meduri et al., 2013; Zhao et al., 2014; Feng et al., 2016), and was investigated using ^13^C and ^19^F NMR spectra, indicating that the controlled formation of the so-called “semi-ionic” C-F bond in the GF_x_ is the most critical factor to achieve high power densities with high energy densities. With an optimal semi-ionic C-F bond ratio, our GF_x_ showed extraordinary performance with a power density of 21,460 W kg^−1^ and an energy density of 1,073 Wh kg^−1^ when the x in GF_x_ equals 0.8, superior to most of the previously reported fluorinated carbons (Mickelson et al., 1998; Lam and Yazami, 2006; Shulga et al., 2007; Yazami et al., 2007; Zhang W. et al., 2010; Fulvio et al., 2011; Groult et al., 2011; Guérin et al., 2012; Damien et al., 2013; Meduri et al., 2013; Sun et al., 2014; Zhao et al., 2014; Feng et al., 2016; Li et al., 2016; Wang et al., 2016). In addition, our approach applied to synthesize GF_x_ is facile, and easy for scale-up, exhibiting very promising practical application.

## Experimental

### Preparation of fluorinated graphene

Fluorinated graphenes were prepared by a one-step gas-phase fluorination of RGO as described in previous work (Yue et al., 2013; Shao et al., 2016). Graphene oxides were prepared using Hummers method (Hummers and Offeman, 1958), which was subjected to thermal reduction for 10 h under a H_2_ flow (5 vol. % in Ar) of 20 sccm at 1,000°C. The resulting RGO was employed to prepared fluorinated graphene materials at 400, 430, and 460°C in fluorinating gas atmosphere for 12 h, obtaining GF_0.5_, GF_0.8_, and GF_1.1_, respectively. The F/C atomic ratios in these samples were determined by quantitative ^13^C NMR: F/C = (S_CF_ + 2 × S_CF2_)/(S_C_+ S_CF2_+ S_CF_), where S is the integrated intensities of the 13C NMR peaks.

### Material characterization

X-ray powder diffraction (XRD) technique was employed to characterize phases of as-prepared materials, using Cu K_α_ radiation (1.54178 Å) on a Miniflex600 (Rigaku, Japan) instrument. XRD patterns were collected with a step of 0.0167°, and 20 s per step. ^13^C and ^19^F magic angle spinning (MAS) NMR experiments were performed on Bruker 600 MHz AVANCE III spectrometer using Hahn-echo pulse under the spinning frequencies of 12 and 60 kHz, respectively. Recycle delays of 60 and 20 s were applied for complete relaxation of excited magnetization for the acquisition of quantitative ^13^C and ^19^F NMR spectra. The chemical shifts of ^13^C and ^19^F were referenced to diamantine (38.6 ppm) and LiF (−204 ppm). X-ray photoelectron spectroscopy (XPS) of the samples was measured by an ESCALAB 250Xi spectrometer (Thermo Fisher). SEM images were performed on scanning electron microscopy (SEM) (ZEISS). The transmission electron microscopy (TEM) and high-resolution TEM (HRTEM) analysis were performed on Tecnai F20 (FEI, US), operating at 200 kV. Nitrogen adsorption/desorption isotherms and Brunauer-Emmett-Teller (BET) surface area were performed on a Quantachrome instrument Autosorb-iQ.

### Electrochemical test

The cathode was prepared by mixing 80 wt.% fluorinated graphene, 10 wt.% acetylene black, and 10 wt.% poly (vinylidene fluoride) (PVDF). Aluminum disks were employed as current collectors and the active materials on the Al disks are between 1.5 and 2 mg cm^−2^. A lithium metal disk was used as a counter electrode, and electrolytes were 1 M LiPF_6_ dissolved in ethylene carbonate/dimethyl carbonate (1:1 volume ratio). Discharge tests were performed at various currents with a cutoff voltage of 1.5 V by a LAND CT2001A battery test system at 25°C.

## Results and discussion

### Electrochemical performance

It is well known that the fluorine content in fluorinated carbon significantly affects the electrochemical performance. A low fluorine content results in a good power density but relatively low energy density. To achieve excellent power densities with high energy densities, we prepared fluorinated graphene (GF_x_) with various F content and investigated the factors influencing the electrochemical performance. The F content in the GF_x_ was controlled by varying the temperatures during the fluorination; for example, GF_0.5_, GF_0.8_, and GF_1.1_ were obtained at 400, 430, and 460°C, respectively.

**Figure 1:**
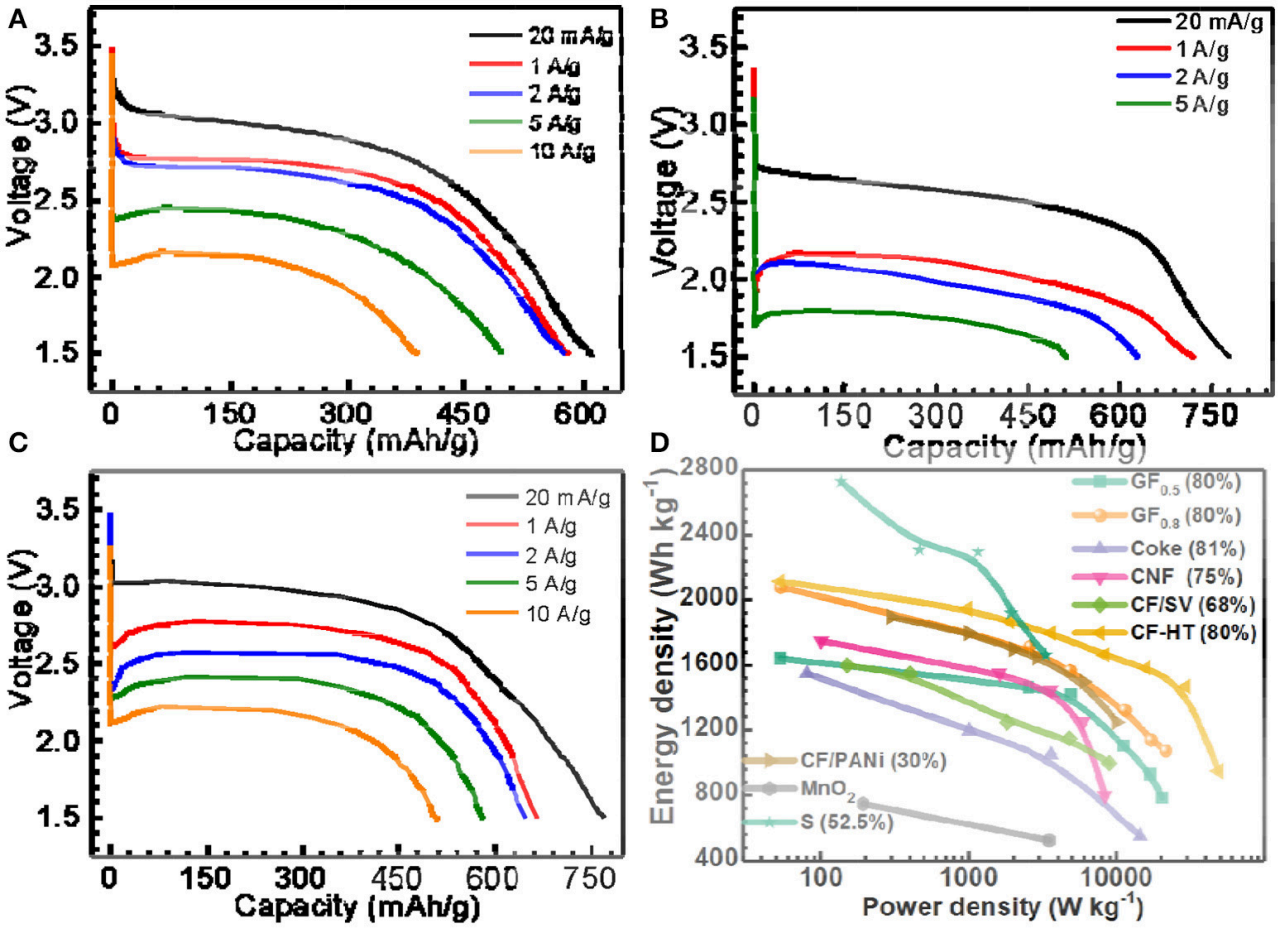
Selected discharge curves of **(A)** GF_0.5_, **(B)** GF_1.1_, **(C)** GF_0.8_ at different current densities, and **(D)** energy density vs. power density plots (Ragone plots) of the as-prepared GFx, typical fluorinated carbons, MnO_2_ and sulfur materials for primary Li batteries, in which the numbers in brackets indicate the mass percentages of active materials on the electrodes. CNF, SV, CF-HT, and PANi represent fluorinated carbon nanofiber, silver vanadate, fluorinated carbon with hydrothermal treatment, and polyaniline, respectively.

When the x in GF_x_ equals 0.5, as shown in Figure 1A, the GF_0.5_ material delivered a capacity of 615 mAh g^−1^ (98.7% of its theoretical capacity) with a discharge plateau at ~2.9 V at a low current density of 20 mA g^−1^ (~1/32C, 1C = 623 mA g^−1^ for GF_0.5_). When the current densities increased to 1 A g^−1^, the capacity of the GF_0.5_ decreased slightly to around 580 mAh/g. Even if the current densities of 5 and 10 A g^−1^ were applied, the GF_0.5_ was still able to deliver 76.9 and 62.4%, respectively, of its theoretical capacity, exhibiting very excellent rate capability. To the best of our knowledge, this is the best rate capability among the untreated fluorinated carbon materials reported in the literatures (Giraudet et al., 2006; Yazami et al., 2007; Zhang W. et al., 2009; Fulvio et al., 2011; Dubois et al., 2012).

With the impressive rate capability, the specific capacity of the GF_0.5_ is yet to be satisfied due to its low theoretical capacity (623 mAh g^−1^). In contrast, the theoretical capacity of the GF_1.1_ is up to 896 mAh g^−1^. As shown in Figure 1B, the GF_1.1_ depicts a capacity of 779 mAh g^−1^, which is 1.34 times of that of the GF_0.5_ at 20 mA g^−1^. However, at high current densities, the voltage plateaus of the GF_1.1_ decreased much more significantly compared with those of the GF_0.5_, suggesting much higher polarization for the GF_1.1_ material. For example, at 5 A g^−1^ the voltage plateau of the GF_1.1_ dropped to 1.77 V with a capacity of 511 mAh g^−1^ that is only 57% of its theoretical capacity. Furthermore, the GF_1.1_ could not work at 10 A g^−1^ at all.

The relatively poor rate capability of the GF_1.1_ is associated with its poor electrical conductivity, which needs to be addressed. Therefore, the way to gain high power densities while retaining high energy densities is to prepare CF_x_ with high F content without compromising the electrical conductivity. To achieve this goal, instead of with complicated surface treatments (Groult et al., 2011; Reddy et al., 2013; Dai et al., 2014), we took the full advantage of high electrical conductivity of the semi-ionic CF bond in CF_x_ (discussed in detail below). In other words, we made great effort to improve the F content within the limit without losing the semi-ionic bonds in the CF_x_. With an optimal F content, the as-designed GF_0.8_ showed high capacity with excellent rate performance (Figure 1C). The specific capacity of the GF_0.8_ was 770 mAh g^−1^, 97.8% of its theoretical capacity, at 20 mA g^−1^ (about 1/39C, 1C = 788.2 mA g^−1^ for GF_0.8_). When the current densities enhanced to 10 A g^−1^ (~12C), the GF_0.8_ exhibited an extraordinary power density of 21,460 W kg^−1^ with a capacity of 511 mAh g^−1^, corresponding to 64.9% of the theoretical capacity and an energy density of 1,073 Wh kg^−1^.

To understand the electrochemical performance of GF_x_ electrode, we compared the kinetic properties of GF_0.5_, GF_0.8_, and GF_1.1_ by electrochemical impedance spectroscopy (EIS) measurement. To exclude the effect of conductive carbon, we prepared the electrodes with the GF_x_ materials without addition of carbon black. In the absence of conductive carbon, however, the impedances of the fresh electrodes were too extremely high to obtain accurate results for comparison (Figure S1A) at open-circuit potentials. Therefore, 1% of the theoretical capacity was discharged to reasonably compare the impedances of the CF_x_ electrodes. As illustrated in Figure S1B, the charge-transfer resistance (R_ct_) of the GF_1.1_ is 2,500 Ω, much higher than that of the GF_0.5_ (~1,400 Ω) and the GF_0.8_ (~1,600 Ω).

Ragone plots were employed to depict the advanced electrochemical performance of the GF_0.8_ (Figure 1D), which is superior to most of the fluorinated carbon and other primary batteries in the literature (Table S1) (Giraudet et al., 2006; Lam and Yazami, 2006; Yazami et al., 2007; Zhang W. et al., 2009; Fulvio et al., 2011; Meduri et al., 2011; Dubois et al., 2012; Guérin et al., 2012; Wang et al., 2012; Adcock et al., 2013; Reddy et al., 2013; Dai et al., 2014, 2017; Sideris et al., 2014; Li and Feng, 2015; Liang et al., 2015; Zhang et al., 2015; Li et al., 2016; Zhu et al., 2017). For example, Lam and Yazami prepared fluorinated coke materials, which showed a maximum power density of about 14,400 W kg^−1^ with energy density of 500 Wh kg^−1^ (Lam and Yazami, 2006). Sulfur material typically display greater energy density but much lower power density due to awfully low electrical conductivity (~10^−30^ S cm^−1^), for which a large amount of carbon has to been applied during electrode preparation (Manthiram et al., 2015; Pang et al., 2015).

### Bonding characteristics of GF_x_ materials

As mentioned above, we achieve high energy densities with excellent power densities by the full use of the advantage of semi-ionic C-F bonds in the CF_x_. Ionic, semi-ionic, and covalent C-F bonds are the three types of C-F bonds in the CF_x_. Ionic C-F bonds are typically only formed when x in CF_x_ is very small (e.g., <0.05 for graphite; Amine and Nakajima, 1993; Nansé et al., 1997; Giraudet et al., 2006), which is not useful in CF_x_/Li batteries because of the very low capacity. Covalent C-F bonds possess the characteristics of the sp^3^ C with the F-C-C angles larger than 90° and the neighboring C-C bond length of ~0.153 nm (Sato et al., 2004; Figure S2A). High covalent C-F ratio may ruin the conductive network of the conjugated double bonds, exhibiting insulating property (electrical conductivity lower than 10^−15^ S cm^−1^; Sato et al., 2004).

In contrast, semi-ionic C-F bonds are essentially covalent, with which, however, the conjugated C-C bonds are preserved between carbon atoms unbounding to fluorine with the F-C-C angle of 90° and the neighboring C-C bond length of ~0.14 nm (Sato et al., 2004; Figure S2B). Unlike what happens in the presence of covalent C-F bonds, with semi-ionic C-F bonds and a certain proportion of sp^2^ C, CF_x_ may have high electronic conductivity, for example, the electronic conductivity of C_x_F with x around two ranged between 5 × 10^−8^ and 1 × 10^−7^ S cm^−1^ (Sato et al., 2004). In a word, the semi-ionic C-F bonds do not significantly degrade the electrical conductivity of the CF_x_ (Mallouk and Bartlett, 1983; Amine and Nakajima, 1993; Sato et al., 2004; Zhang W. et al., 2010), which can be employed to gain high power densities with high energy densities.

#### Solid-state ^19^F and ^13^C NMR spectra

**Figure 2:**
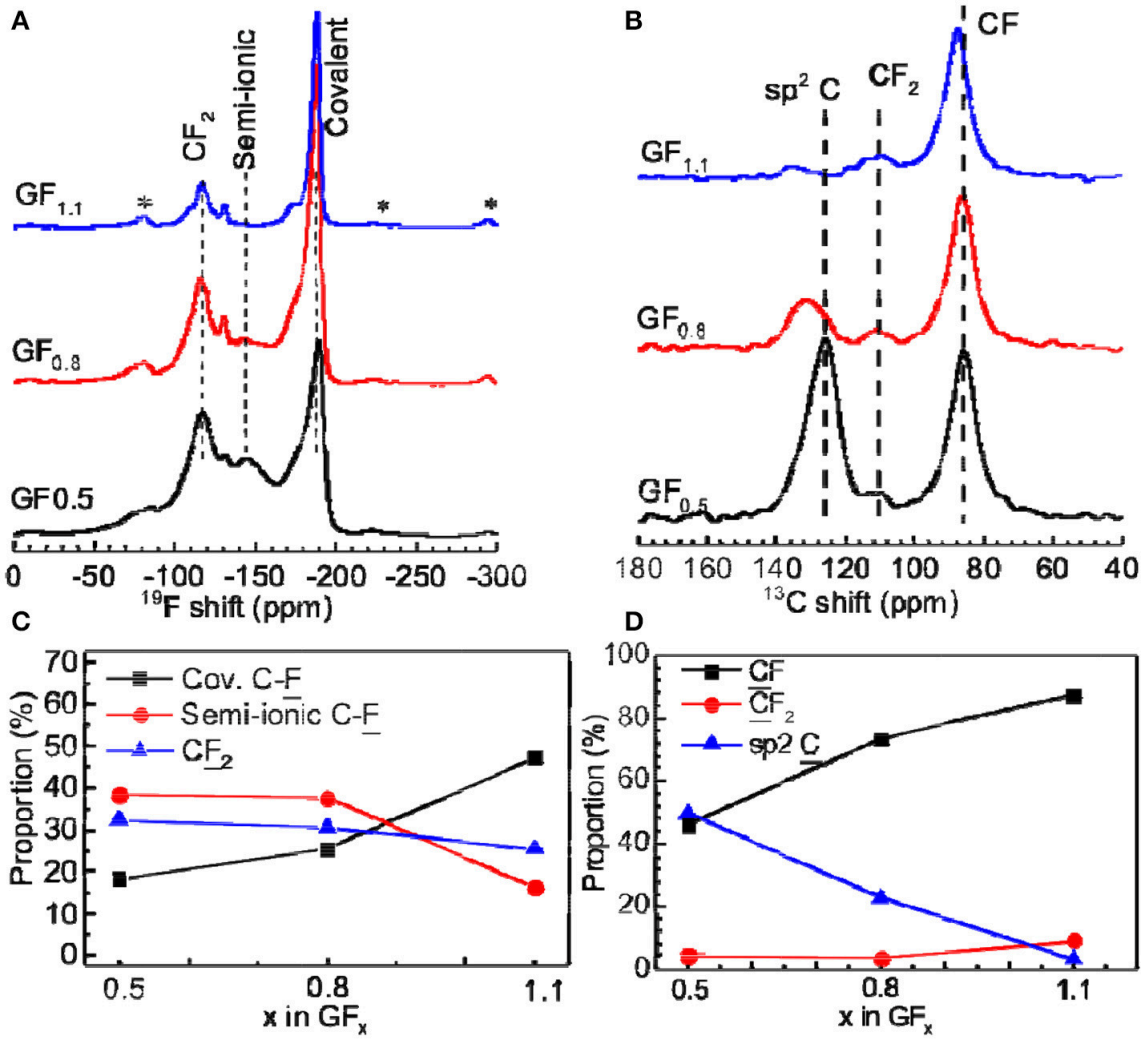
**(A)** ^19^F and **(B)** ^13^C MAS NMR spectra of GF_x_ acquired at spinning frequencies of 60 and 12 kHz, respectively. The asterisks indicate the spinning sidebands. **(C,D)** Integral area proportions of fluorine and carbon with different bonding properties from ^19^F and ^13^C NMR spectra, respectively.

^19^F NMR spectra were employed to distinguish covalent and semi-ionic C-F bonds. Figure 2A shows that the ^19^F MAS NMR spectra acquired at a spinning frequency of 60 kHz, in which the ^19^F resonances consist of three parts. Resonance peaks located between −80 and −135 ppm belong to the signal of CF_2_ groups. Resonance peaks located between −140 and −180 ppm were assigned to the signal of the semi-ionic CF group, while the peaks located between −185 and −189 ppm were assigned to the signal of the covalent CF group (Panich et al., 1997; Krawietz and Haw, 1998; Giraudet et al., 2007; Leifer et al., 2010; Ahmad et al., 2013).

The semi-ionic C-F bond ratios in the GF_x_ were determined by fitting of ^19^F NMR spectra (Figure S3). As shown in Figure 2C, the semi-ionic bond ratio in GF_0.5_ is 38.5%, which slightly decreases to 37.7% when the x in GF_x_ increases to 0.8. Beyond that point, the semi-ionic bond ratio in the GF_x_ dramatically dropped (e.g., 16.1% in GF_1.1_), which is consistent with the facts that the rate capability of the GF_0.8_ is similar with that of the GF_0.5_, but much better than that of the GF_1.1_. For example, at 5 A g^−1^, the energy densities of the GF_0.5_, GF_0.8_, and GF_1.1_ are 10,856, 11,352, and 898 Wh kg^−1^, respectively (Figure 1).

Another possible reason for poor electrical conductivity related to the low semi-ionic C-F bond ratio is the high F content resulting in low content of the sp^2^ hybridized C. Figure 2B exhibits the ^13^C NMR spectra, in which the resonance peaks at around 130, 111, and 87 ppm are associated with the sp^2^ C, CF_2_, and CF, respectively. Their contents were calculated by fitting the peaks (Figure S4). The sp^2^ C contents in the GF_0.5_, GF_0.8_, and GF_1.1_ are calculated to be 49.8, 22.8, and 3.4%, respectively (Figure 2D). When the sp^2^ C content goes down to too low value (e.g., 3.4% in the GF_1.1_), the electrical conductivity is compromised (Yue et al., 2013), resulting in a poor rate capability.

Based on the ^19^F and ^13^C NMR analysis, we can conclude that with the increasing F content in GF_x_, the sp^2^ C ratio decreases while the semi-ionic C-F bond ratio remains unchanged until the critical x of 0.8, beyond which the electron-transfer ability of sp^2^ C is compromised. This matches very well the electrochemical performance of GF_0.5_, GF_0.8_, and GF_1.1_, namely, the GF_0.5_ and GF_1.1_ depicted high power densities and high capacities, respectively, but the GF_0.8_ exhibited the optimal electrochemical performance (21,460 W kg^−1^ and 1,073 kWh kg^−1^).

#### XPS spectra

**Figure 3:**
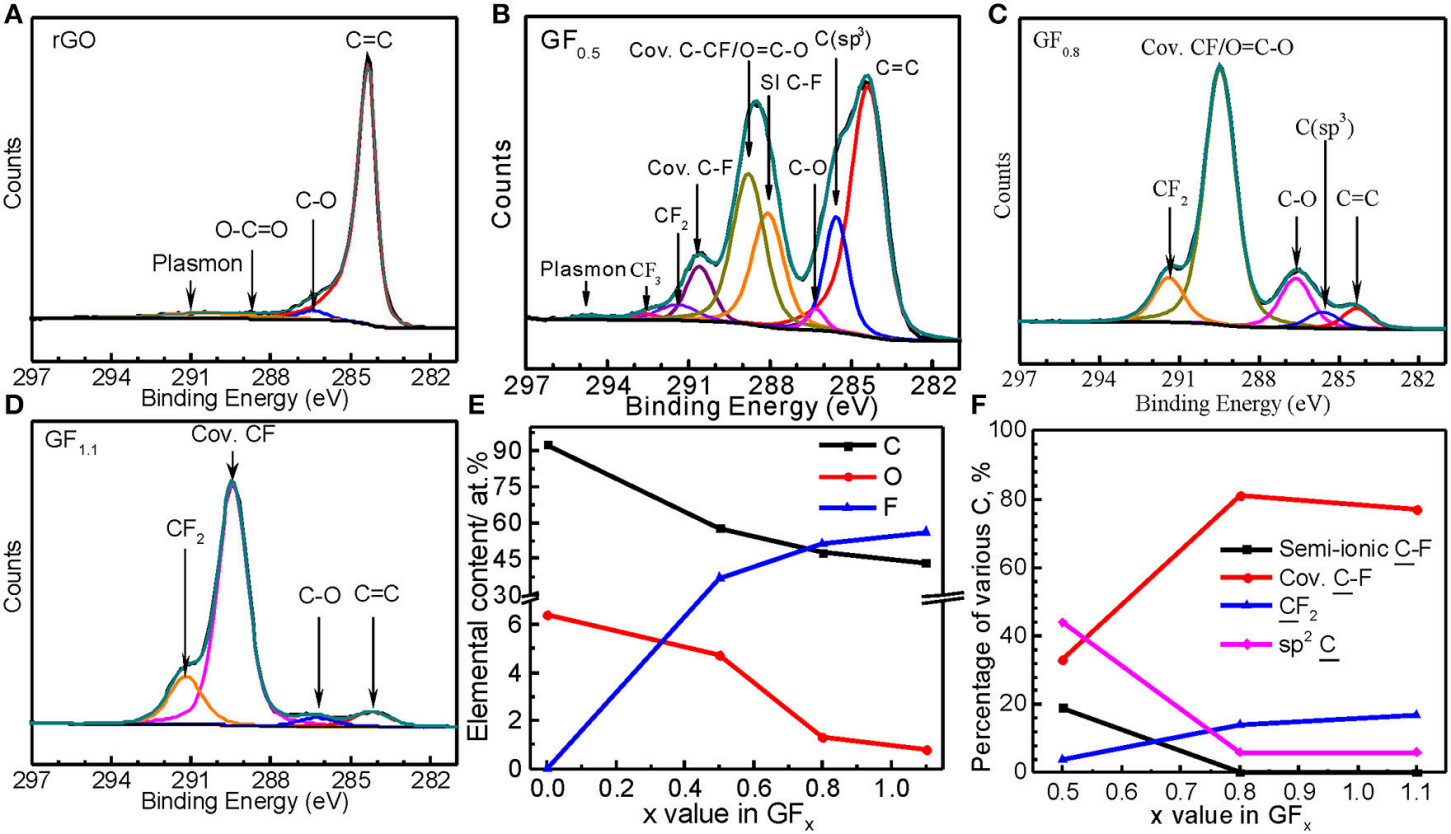
XPS C1s spectra of **(A)** RGO, **(B)** GF_0.5_, **(C)** GF_0.8_, and **(D)** GF_1.1_, and quantitative analysis results from XPS survey spectra **(E)** and C1s spectra **(F)**.

Semi-ionic C-F bond ratios in CF_x_ also have been analyzed using X-ray photoelectron spectroscopy (XPS) spectra (Doniach and Sunjic, 1970; Tressaud et al., 1996; Nansé et al., 1997; Leiro et al., 2003; Park et al., 2008). We therefore conducted XPS analysis for our GF_x_ materials for comparison. As shown in Figure 3A, the carbon in the RGO was mainly composed of C = C sp^2^ bonds (284.4 eV) and minor bonds C-O-C (~286.5 eV) and O-C = O (~288.6 eV). In the GF_0.5_ (Figure 3B), the binding energies at 288.0 and 288.8 eV were assigned to the semi-ionic and the covalent CF bonds, respectively (Tressaud et al., 1996; Nansé et al., 1997; Wang et al., 2014).

In contrast, the semi-ionic C-F bonds were barely detected by XPS C1s spectra for the GF_0.8_ and GF_1.1_ (Figures 3C,D), which are consistent with the results from the XPS F1s spectra (Figure S5); no semi-ionic C-F bond was detected in the GF_0.8_ and GF_1.1_. In other words, the C-F bond on the surface of the GF_0.8_ and GF_1.1_ materials were mainly covalent. Apparently, these results are inconsistent with those from NMR, where the semi-ionic C-F ratios in GF_0.8_ and GF_1.1_ are much higher. This is because that the surface of the GF_x_ is subjected to more attack than the bulk during fluorination, resulting in higher fluorinating levels on the GF_x_ surface than in the bulk. The semi-ionic C-F in GF_0.8_ and GF_1.1_can be detected by NMR but not by XPS because that is for surface analysis while the former obtains bulk information. As a conclusion, NMR is more suitable for analyzing semi-ionic C-F bond ratio in CF_x_ than XPS (Figures 3E,F).

Besides the semi-ionic C-F bond ratio, other factors that may influence the electrochemical performance of the GF_x_ were also investigated, including structure, morphology, and surface area.

### XRD patterns and morphology features

#### XRD patterns and TEM images

XRD patterns of fluorinated graphene materials were shown in Figure S6. Two peaks centered at around 25.4 and 43.2° were observed for the RGO, which correspond with the 002 and 100 reflections of graphitic carbon, respectively. After fluorination, the 002 reflection decreased significantly while a new peak at ~15° developed for the three fluorinated samples, which may be assigned to the 001 plane of fluorinated phase (Hamwi, 1996; Meduri et al., 2013) or the expanded 002 plane (Zhang et al., 2015). The layer thickness of the RGO increased from ~0.4 to ~0.6 nm after fluorination as indicated by HRTEM (Figures S7–S9), which is in accordance with results from XRD patterns (Figure S6). Note also that the 100 reflections shifted to lower angle with increasing fluorination levels, indicating an increasing C-C in-plane length, which is consistent with the trend of fluorinated graphite (Guérin et al., 2004). Theoretically, increased interplanar distances in the GF_x_ may facilitate the intercalation of lithium ions. However, considering the fact that GF_1.1_ shows a poor rate capability than the GF_0.8_, the increasing of *d*-spacing does not offer enough help the GF_1.1_ to gain excellent power density.

#### SEM images

**Figure 4:**
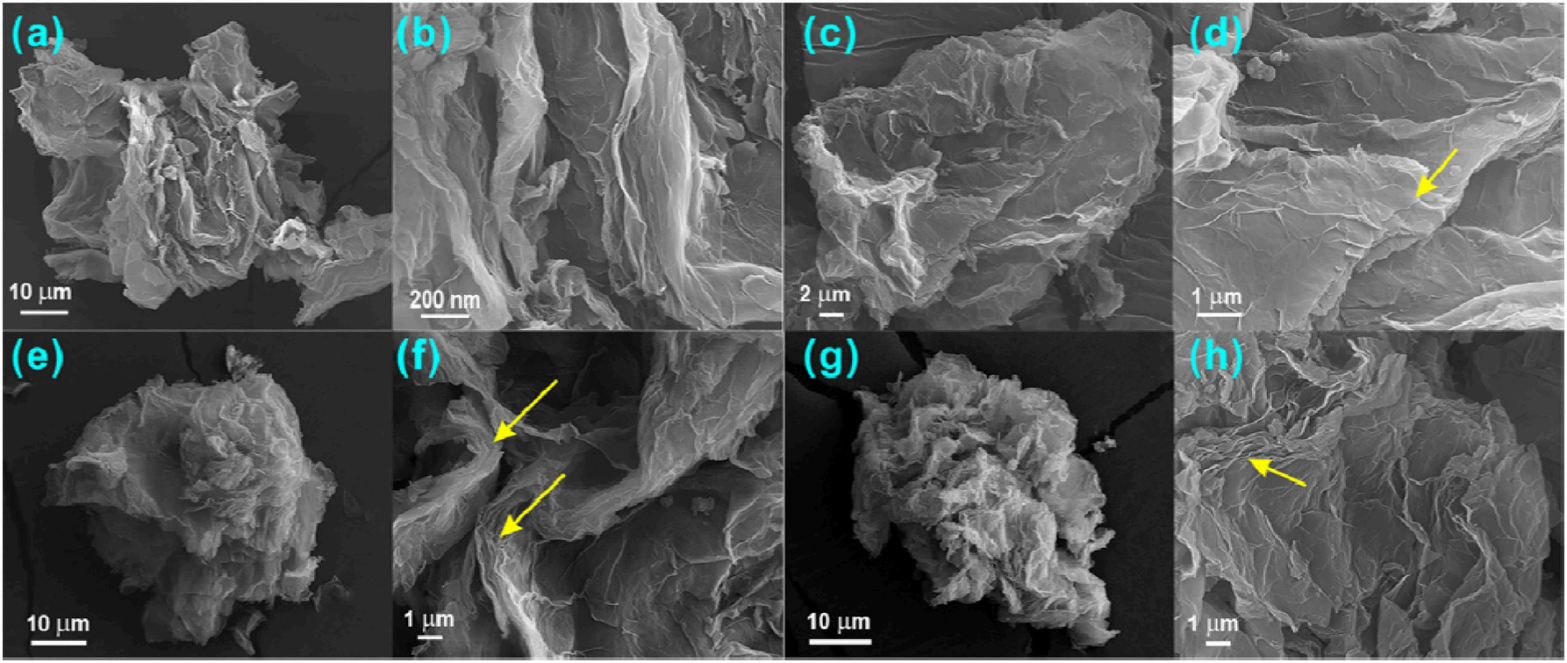
SEM images of **(a,b)** RGO, **(c,d)** GF_0.5_, **(e,f)** GF_0.8_, and **(g,h)** GF_1.1_. The yellow arrows indicate the lamellar structure of fluorinated graphenes.

Figure 4 showed the SEM images of the RGO and GF_x_ materials, indicating their secondary particle sizes larger than 10 microns due to the aggregation of GO upon drying. Interestingly, compared with pristine RGO, the fluorinated graphene materials exhibit clear lamellar architectures that were marked by the yellow arrows in Figures 4d,f,h; especially in GF_0.8_ and GF_1.1_, some fluorinated graphene layers seem to be peeled off. Although fluorination introduces F into the interlayers of C, the *d*-spacing of 002 facet only expands from ~0.33 to ~0.6 nm. Therefore, we believe the splitting of graphene layers is not due to the F expansion, but to the attack by the fluorinating gas during the fluorination at high temperatures. As a matter of fact, the GF_1.1_ was stripped more severely than the GF_0.8_ and much more than the GF_0.5_, which agrees well with their preparation temperatures. The splitting of graphene layers might facilitate the diffusion of solvated lithium ions compared with other types of fluorinated carbon materials (Meduri et al., 2013), thereby enhancing the rate capability of GF_x_. However, the power density of the GF_1.1_ is poorer than that of the GF_0.8_, which is inconsistent with the lager expanded spaces. Therefore, the effect of morphology change on the rate performance is inferior to the semi-ionic C-F bond ratio in the GF_x_.

#### BET results

**Figure 5:**
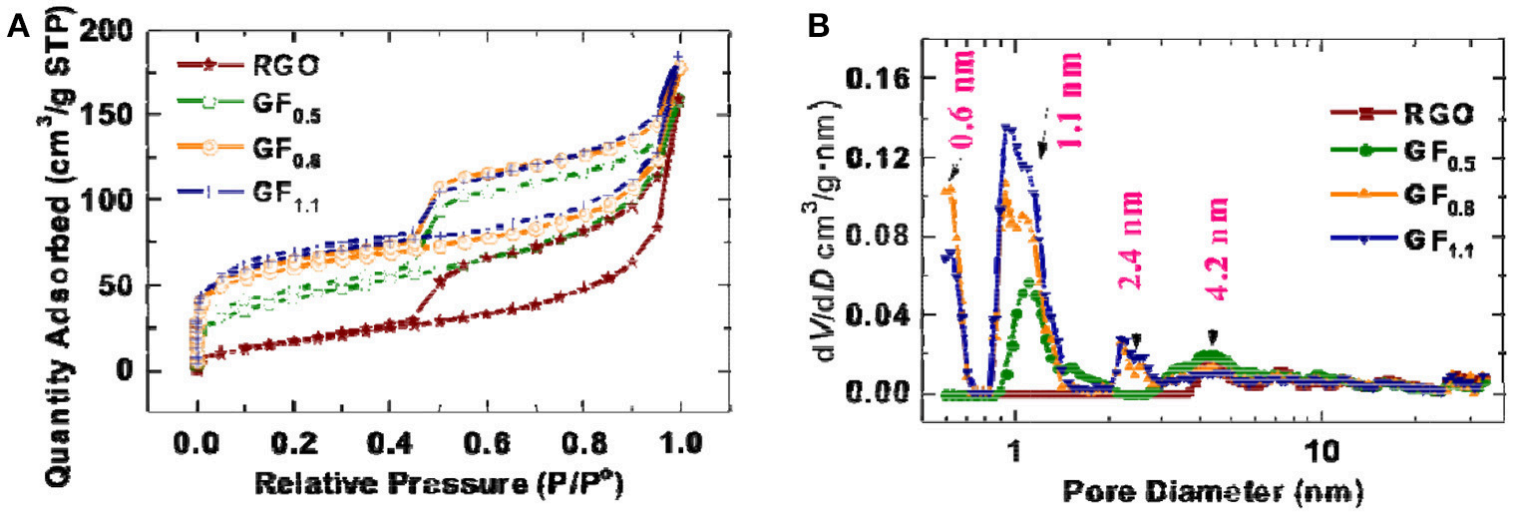
**(A)** Nitrogen adsorption-desorption isotherm and **(B)** pore size distribution of pristine RGO and the fluorinated graphene materials.

During fluorination, the surface area may change, thereby contributing to the improved rate performance. Therefore, the specific surface areas of the pristine RGO and the fluorinated graphene materials were analyzed using Brunauer–Emmett–Teller (BET) method. Figure 5A exhibits the nitrogen adsorption-desorption isotherm; accordingly, the BET specific surface areas for the RGO, GF_0.5_, GF_0.8_, and GF_1.1_ were measured to be 70.2, 152.4, 215.8, 238.6 m^2^ g^−1^, respectively. The relatively small specific area of the RGO is due to the relatively thick graphene, which is confirmed by HRTEM (Figure S7). The thickness of the RGO is up to 20 layers from the HRTEM observation.

After fluorination, the specific area increased, which is consistent with the SEM observation, in which the fluorinated graphenes exhibit more lamellar architectures, compared with the pristine RGO (Figure 5). The pore sizes of the as-prepared fluorinated graphenes were analyzed based on a quenched solid density functional theory (QSDFT) kernel applied to the adsorption branch using a slit/cylindrical pore model (Ravikovitch and Neimark, 2006; Gor et al., 2012). As shown in Figure 5B, the RGO possesses mesopores (~4.2 nm) without any micropores. In contrast, after fluorination, micropores were developed at 1.1 nm for the GF_0.5_ and an additional micropore size was observed at 0.6 nm for the GF_0.8_ and GF_1.1_. Although the increased pores and surface areas might facilitate lithium ion transfer, the higher surface the as-obtained fluorinated graphenes did not result in a better rate capability. Therefore, the surface area of the fluorographenes is not the determining factor affecting the power densities of the fluorinated graphenes.

## Conclusions

Fluorinated graphenes were prepared using one-step gas fluorination of RGO at elevated temperatures. The impacting factors, including semi-ionic C-F ratio, sp^2^ C content, structure, morphology, and specific surface area are investigated to gain fluorinated graphenes with high power densities and high energy densities. The semi-ionic C-F ratio in the fluorinated graphene shows the most critical influence on achievement of high rate performance. Thus, by manipulating the semi-ionic C-F proportion in the fluorinated graphene by temperature control, we obtain the optimal x of 0.8 in GF_x_; the GF_0.8_ exhibited a high energy density of 1,073 Wh kg^−1^ and an excellent density of 21,460 W kg^−1^ at a high current density of 10 A g^−1^ (about 12C rate). Compared with those using additional steps (such as C coating and hydrothermal treatment) to improve the rate performance of as-obtained CF_x_, we offer a one-step approach to obtain high energy densities without compromising power densities for preparation of fluorinated carbon, showing very promising practical application.

## Author contributions

The work cannot be completed without kind cooperation of all authors. GZ: Acquired and analyzed the NMR and XPS data; HC: Carried out the material preparation and electrochemical test; XH and HC: Carried out and analyzed the SEM, TEM, and BET analysis; GZ and XH: Wrote the paper and all authors discussed the results and revised the manuscript; HY, GZ, HC, and XH: Proposed the research; HY and CL: Attained the main financial support for the research and supervised all the experiments.

### Conflict of interest statement

The authors declare that the research was conducted in the absence of any commercial or financial relationships that could be construed as a potential conflict of interest. The reviewer XJ, and handling Editor declared their shared affiliation.
