# Supplementary material for: High-Power-Density, High-Energy-Density Fluorinated Graphene for Primary Lithium Batteries
Source: Front Chem. 2018 Mar 9;6:50. doi: 10.3389/fchem.2018.00050 (PMC5854643; doi:10.3389/fchem.2018.00050)
Supplement: Supplementary file 1 [file DataSheet1.DOCX]

**High-power-density, high-energy-density fluorinated graphene for primary lithium batteries**

Guiming Zhong,^a,b,‡^HuixinChen^a,b,‡^ Xingkang Huang,^c,‡^HongjunYue,^a.b,*^CanzhongLu^a.b^

^a^ CAS key Laboratory of Design and Assembly of Functional Nanostructures, and Fujian Provincial Key Laboratory of Nanomaterials, Fujian Institute of Research on the Structure of Matter, Chinese Academy of Sciences, Fuzhou, Fujian 350002, China

^b^ Xiamen Institute of Rare Earth Materials, Haixi institutes, Chinese Academy of Sciences, Xiamen, 361021, China

c Department of Mechanical Engineering, University of Wisconsin-Milwaukee, 3200 North Cramer Street, Milwaukee, Wisconsin 53211, USA







Figure S1. Electrochemical impedance spectroscopy (EIS) measurement at open-circuit potential (a) and after the 1 percent discharge (b) of theoretical capacity without conductive carbon added.


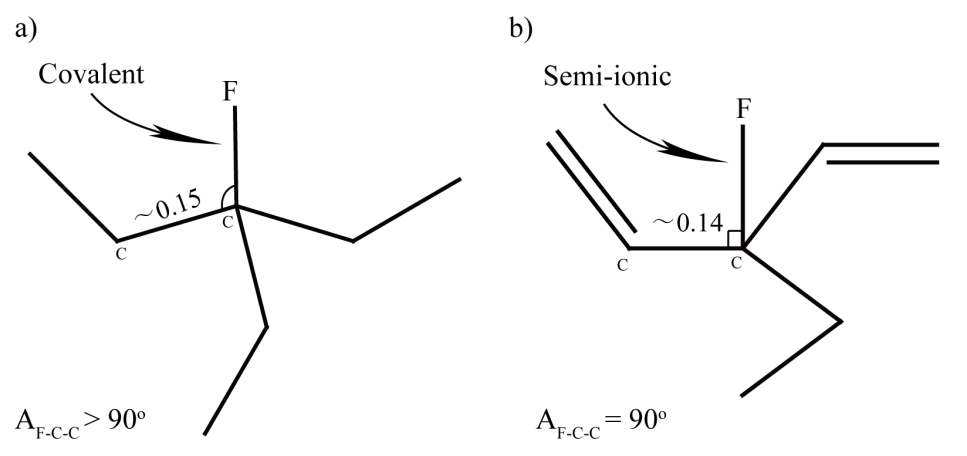


Figure S2. Schematic illustration of covalent and semi-ionic C-F bonding.









Figure S3. Deconvolution plots of ^19^F NMR spectra of GF_0.5_, GF_0.8_ and GF_1.1_ materials.

Figure S3 shows the deconvolution plots of ^19^F NMR spectra. Many peaks were applied for fitting because of variable environments (neighboring groups, bulk or interfacial, phase states, etc.) of CF, CF_2_, and CF_3_ located. Spinning sidebands made the fitting complex, too. Resonance peaks located between -80 and -135 ppm belong to the signal of CF_2_ groups, depending on the phase states and the groups connected.(Pallathadka, et al., 2006; Leifer, et al., 2010) Resonance peaks located between -140 and -180 ppm were assigned to the signal of semi-ionic CF group,(Leifer, et al., 2010; Panich, et al., 1997; R. Krawietz and F. Haw, 1998; Dubois, et al., 2004; Giraudet, et al., 2007; Zhang, et al., 2008; Ahmad, et al., 2013) depending on the bonding properties around the group and bond strength,(Dubois, et al., 2012) while the peaks located between -185 and -189 ppm were assigned to the signal of covalent CF group. Peaks between -50 and -100 ppm were assigned to the signal of CF_3_ and spinning sidebands. Peaks between -200 and -250 ppm were assigned to the spinning sidebands, too. The spinning sidebands and signal of CF_3_ were not included for analysis because that the overlapping peaks may cause fitting errors.









Figure S4. Deconvolution plots of ^13^C NMR spectra of GF_0.5_, GF_0.8_ and GF_1.1_ materials.

Figure S4 shows the deconvolution plots of ^13^C NMR spectra of three fluorinated graphene materials. Three ^13^C resonance signals at around 87, 111 and 130 ppm were assigned to CF, CF_2_, and sp^2^ carbon, respectively.(Leifer, et al., 2010; R. Krawietz and F. Haw, 1998; Giraudet, et al., 2007) Because of the misconvergence of the fitting using three peaks for GF_0.5_, four peaks were applied for the fitting of spectrum of GF_0.5_. The peak between 120 and 150 ppm can be deconvoluted into two peaks located at 125.4 and 130.2 ppm, assigning to the "bulk C" (two or more bonds away from CF) and "interfacial C" (less than two bonds away from CF).(Leifer, et al., 2010; Dubois, et al., 2004) The shift of peak assigning to "interfacial C" indicated an increasing CF group around sp^2^ C atom with increasing fluorine. Due to the fact that the ^13^C shifts of semi-ionic and covalent carbon are almost the same, thus not be distinguishable.(Leifer, et al., 2010) F/C ratios were calculated to be 0.5, 0.8 and 1.1 for three samples according to the following equation(Leifer, et al., 2011): F/C = (S_CF_ + 2×S_CF2_) / (S_C_+ S_CF2_+ S_CF_), where S is the integrated intensities of the ^13^C NMR peaks.









Figure S5. Deconvolution plots of XPS F1s spectra for GF_0.5_, GF_0.8_ and GF_1.1_ materials. The binding energies 686.8, 688 and 689 eV, assigning to the semi-ionic, covalent CF and CF_2_ groups are applied for fitting.


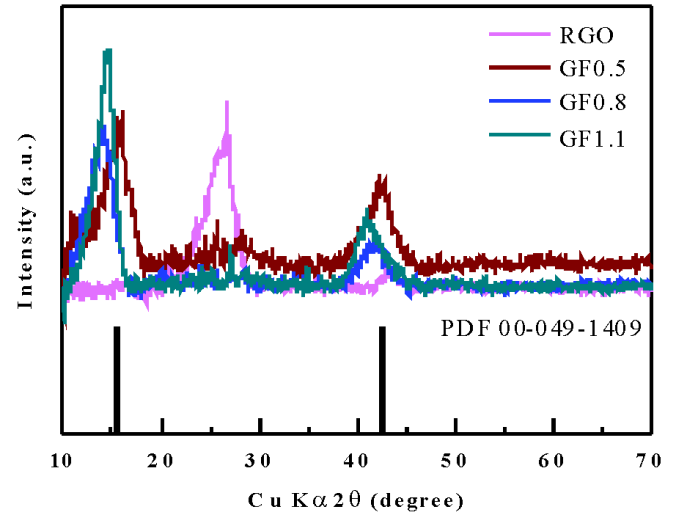


Figure S6 XRD patterns of the pristine RGO and the GF_x_ materials. Two broad peaks at 25.4 and 43.2 ° correspond with the 002 and 111 facets of the RGO, respectively. After fluorination, the 002 peak turned week while a new developed at 15 ° for the GF_0.5_, suggesting the intercalation of F into the layers of the RGO. Further fluorination only slightly increased the layer distance form 0.55 nm (for GF_0.5_) to ~0.6 nm (for GF_0.8_ and GF_1.1_).


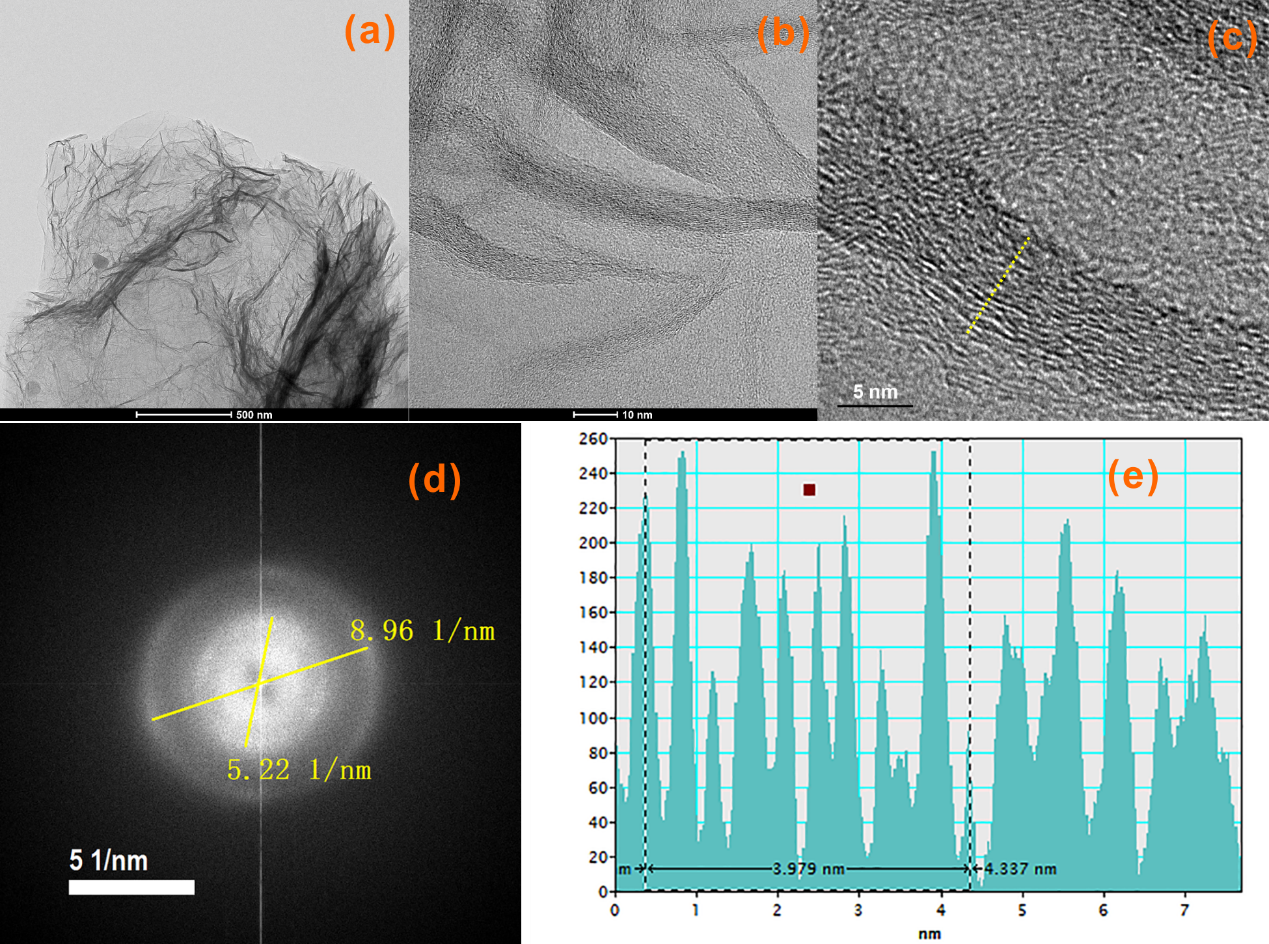


Figure S7 (a) TEM image of RGO, (b, c) HRTEM, (d) FFT image, and (e) profile from the position marked by a yellow dash line in (c).

As shown in Figure S7, the RGO used in this study is a multi-layered RGO, whose thickness is up to 20 layers. The distances of 5.22 and 8.96 1/nm in Figure S7d corresponds with the *d*-spaces of 0.38 and 0.22 nm, which are consistent with the 002 and 100 facets at 25.4 and 43.2 °, respectively, in XRD patterns (Figure S6). The layer distance of the RGO was estimated to be 0.40 nm, which was obtained by averaging the distance of 10 layers of the RGO, as shown in (e).


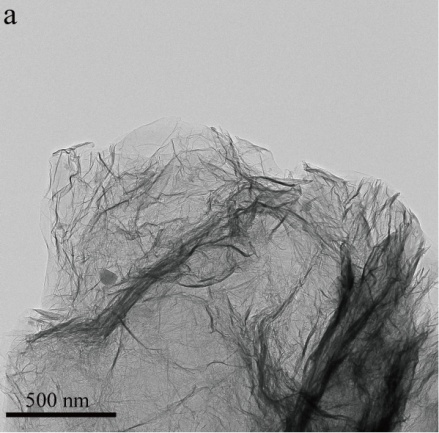

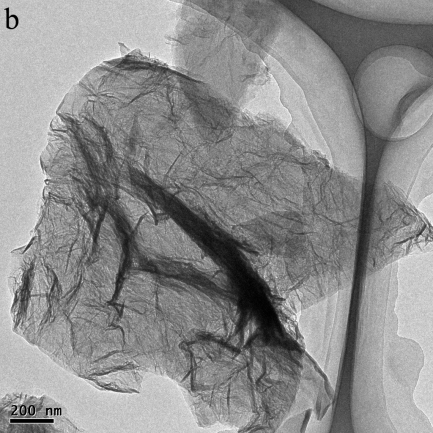


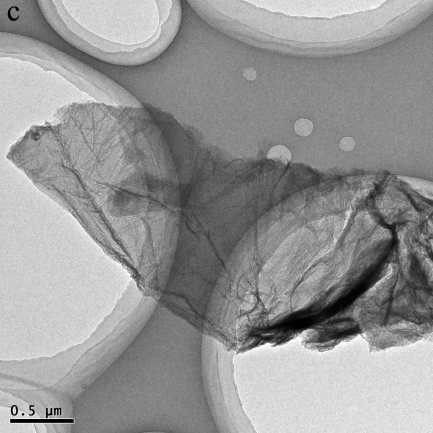

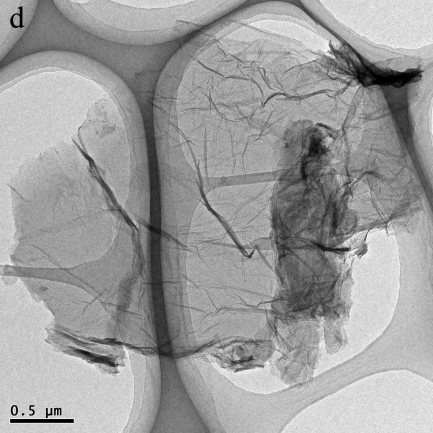


Figure S8 TEM images of a) RGO, b) GF_0.5_, c) GF_0.8_, and d) GF_1.1_.


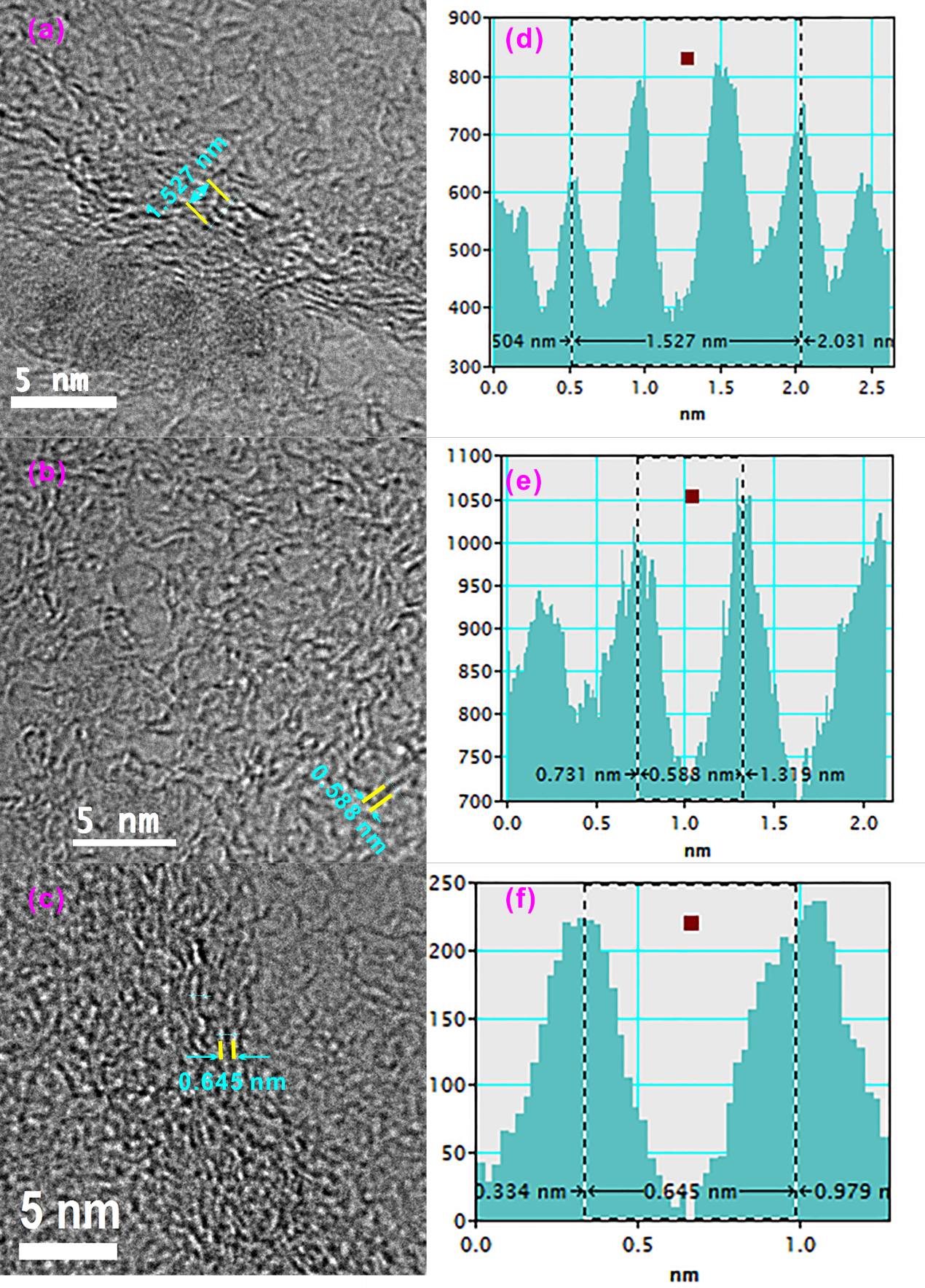


Figure S9. (a-c) HRTEM of the GF_0.5_, GF_0.8_, and GF_1.1_, and their profiles shown in (d-f), respectively.

As shown in Figure S9, the thickness of the GF_0.5_ is at least 8 layers with an average layer thickness of ~0.51 nm. The GF_0.8_ and GF_1.1_ possess fewer layers (typically 2-6 layers) with the layer thicknesses of 0.59 and 0.65 nm, respectively. Note that, apparently, the layer thicknesses from the HRTEM observation are slightly different with those from XRD. This is because the XRD peaks are very broad, corresponding with a broad d-spacing range while HRTEM only observes local values.

Table S1.Comparison of largest power as well as energy density for various fluorinated carbons.

| Carbon or process method | Power(W/kg) | Energy(Wh/kg） | Reference |
| --- | --- | --- | --- |
| Graphene | 21460 | 1073 | This work |
| Coke | 14400 | 550 | (Lam and Yazami, 2006) |
| CNF | 8300 | 800 | (Yazami, et al., 2007) |
| CF/SV | 8900 | 1000 | (Meduri, et al., 2011) |
| CF/PANi | 10000 | 1450 | (Li, et al., 2016) |
| CF-BM | 10000 | 800 | (Reddy, et al., 2013) |
| CF-HT | 54600 | 652 | (Dai, et al., 2017) |

Note that minor errors may exist while reading the data from Figures in the references. CNF, SV, PANi, BM and CF-HT, represent fluorinated carbon nanofibers, silver vanadate, polyaniline, and fluorinated carbon with ball-milling and hydrothermal treatment, respectively.

**References**

Pallathadka, PK. Tay, SS. Tianxi, L and Sprenger, P. (2006). Solid state ^19^F NMR study of crystal transformation in PVDF and its nanocomposites. Polym. Eng. Sci. 46, 1684-1690. doi:<10.1002/pen.20631>

Leifer, ND. Johnson, VS. Ben-Ari, R. Gan, H. Lehnes, JM. Guo, R. et al. (2010). Solid-state NMR studies of chemically lithiated CF_x_. J. Electrochem. Soc. 157, A148-A154. doi:<10.1149/1.3267042>

Panich, AM. Nakajima, T and Goren, SD. (1997). ^19^F NMR study of C-F bonding and localization effects in fluorine-intercalated graphite. Chem. Phys. Lett. 271, 381-384. doi:[10.1016/S0009-2614(97)00450-8](http://dx.doi.org/10.1016/S0009-2614(97)00450-8)

R. Krawietz, T and F. Haw, J. (1998). Characterization of poly(carbon monofluoride) by ^19^F and ^19^F to ^13^C cross polarization MAS NMR spectroscopy. Chem. Commun., 2151-2152. doi:<10.1039/A803252A>

Dubois, M. Guérin, K. Pinheiro, JP. Fawal, Z. Masin, F and Hamwi, A. (2004). NMR and EPR studies of room temperature highly fluorinated graphite heat-treated under fluorine atmosphere. Carbon. 42, 1931-1940. doi:[10.1016/j.carbon.2004.03.025](http://dx.doi.org/10.1016/j.carbon.2004.03.025)

Giraudet, J. Dubois, M. Guérin, K. Delabarre, C. Hamwi, A and Masin, F. (2007). Solid-State NMR study of the post-fluorination of (C_2.5_F)n fluorine−GIC. J. Phys. Chem. B. 111, 14143-14151. doi:<10.1021/jp076170g>

Zhang, W. Dubois, M. Guérin, K. Hamwi, A. Giraudet, J and Masin, F. (2008). Solid-state NMR and EPR study of fluorinated carbon nanofibers. J. Solid State Chem. 181, 1915-1924. doi:[10.1016/j.jssc.2008.03.037](10.1021/jp076170ghttp:/dx.doi.org/10.1016/j.jssc.2008.03.037)

Ahmad, Y. Dubois, M. Guérin, K. Hamwi, A. Fawal, Z. Kharitonov, AP. et al. (2013). NMR and NEXAFS study of various graphite fluorides. J. Phys. Chem. C. 117, 13564-13572. doi:<10.1021/jp401579u>

Dubois, M. Guérin, K. Zhang, W. Ahmad, Y. Hamwi, A. Fawal, Z. et al. (2012). Tuning the discharge potential of fluorinated carbon used as electrode in primary lithium battery. Electrochim. Acta. 59, 485-491. doi:[10.1016/j.electacta.2011.11.015](http://dx.doi.org/10.1016/j.electacta.2011.11.015)

Leifer, N. Smart, M. Prakash, G. Gonzalez, L. Sanchez, L. Smith, K. et al. (2011). ^13^C solid state NMR suggests unusual breakdown products in SEI formation on lithium ion electrodes. J. Electrochem. Soc. 158, A471-A480. doi: 10.1149/1.3559551

Lam, P and Yazami, R. (2006). Physical characteristics and rate performance of (CF_x_)n (0.33 < x < 0.66) in lithium batteries. J. Power Sources. 153, 354-359. doi:[10.1016/j.jpowsour.2005.05.022](https://doi.org/10.1016/j.jpowsour.2005.05.022)

Yazami, R. Hamwi, A. Guérin, K. Ozawa, Y. Dubois, M. Giraudet, J. et al. (2007). Fluorinated carbon nanofibres for high energy and high power densities primary lithium batteries. Electrochem. Commun. 9, 1850-1855. doi:[10.1016/j.elecom.2007.04.013](http://dx.doi.org/10.1016/j.elecom.2007.04.013)

Meduri, P. Chen, H. Chen, X. Xiao, J. Gross, ME. Carlson, TJ. et al. (2011). Hybrid CF_x–_Ag_2_V_4_O_11_ as a high-energy, power density cathode for application in an underwater acoustic microtransmitter. Electrochem. Commun. 13, 1344-1348. doi:[10.1016/j.elecom.2011.08.006](http://dx.doi.org/10.1016/j.elecom.2011.08.006)

Li, L. Zhu, L. Pan, Y. Lei, W. Ma, Z. Li, Z. et al. (2016). Integrated polyaniline-coated CF_x_ cathode materials with enhanced electrochemical capabilities for Li/CF_x_ primary battery. Int. J. Electrochem. Sci. 11, 6838-6847.

Reddy, MA. Breitung, B and Fichtner, M. (2013). Improving the energy density and power density of CFx by mechanical milling: a primary lithium battery electrode. ACS Appl. Mater. Inter. 5, 11207-11211. doi:<10.1021/am403438m>

Dai, Y. Fang, Y. Cai, S. Wu, L. Yang, W. Yan, H. et al. (2017). Surface modified pinecone shaped hierarchical structure fluorinated mesocarbon microbeads for ultrafast discharge and improved electrochemical performances. J. Electrochem. Soc. 164, A1-A7. doi:<10.1149/2.0451614jes>
